# The Theory of Hyperlipidemic Memory of Type 1 Diabetes

**DOI:** 10.3389/fendo.2022.819544

**Published:** 2022-03-31

**Authors:** Benjamin Udoka Nwosu

**Affiliations:** ^1^Division of Endocrinology, Department of Pediatrics, Zucker School of Medicine at Hofstra/Northwell, New Hyde Park, NY, United States; ^2^Division of Endocrinology, Department of Pediatrics, University of Massachusetts Medical School, Worcester, MA, United States

**Keywords:** type 1 diabetes, type 2 diabetes, adults, honeymoon phase, partial clinical remission, cardiovascular disease risk, dyslipidemia, hyperlipidemia

## Abstract

A literature search was conducted to identify publications addressing the early phases of lipid phenotypes in children and adults with either type 1 diabetes or type 2 diabetes. Medline, EMBASE, and Ovid were searched using the following search terms: *clinical remission, partial remission, partial clinical remission, honeymoon phase, C-peptide, type 1 or 2 diabetes, children, pediatric type 1 or 2 diabetes, and paediatrics type 1 or 2 diabetes, adults, adult type 1 or type 2 diabetes.*

Partial clinical remission (PR) of type 1 diabetes (T1D) is characterized by continued endogenous production of insulin and C-peptide following the diagnosis and the introduction of exogenous insulin therapy. PR is associated with improved glycemic control and reduced prevalence of diabetes complications. The theory of hyperglycemic memory was proposed to explain this concept of improved glycemic outcomes in remitters (those who experienced PR) versus non-remitters (those who did not experience PR). However, this theory is incomplete as it does not explain the dichotomy in early lipid phenotypes in T1D based on PR status, which is an understudied area in diabetology and lipidology. To fill this knowledge gap, we propose the Theory of Hyperlipidemic Memory of T1D. This theory is premised on our 5-year research on early post-diagnostic dichotomy in lipid phenotypes between remitters and non-remitters across the lifespan. It provides a more rigorous explanation for the differences in lifelong atherosclerotic cardiovascular disease (ASCVD) risk between remitters and non-remitters. We conducted 4 clinical studies in pediatric and adult subjects with diabetes mellitus to characterize the particulars of the hyperlipidemic memory. In the first investigation, we explored the impact of the presence or absence of PR on lipid parameters in children and adolescents with T1D. In the second, we investigated whether pubertal maturation influenced our findings in T1D; and whether these findings could be replicated in healthy, non-diabetic children and adolescents. In the third, we leveraged our findings from T1D and controls to investigate the mechanisms of early lipid changes in T2D by comparing the earliest lipid phenotype of subjects with type 2 diabetes (T2D) to those of remitters, non-remitters, and controls. In the fourth, we investigated the impact of PR on the earliest lipid phenotypes in adults with T1D and compared these early lipid data to those of T2D subjects and controls. This body of work across the lifespan in children, adolescents, and adults supports the Theory of Hyperlipidemic Memory. This new theory clarifies why PR largely determines the risks for early-phase dyslipidemia, mid-term microvascular disease risk, and long-term ASCVD risk in subjects with T1D.

## Introduction

Diabetes mellitus affects 34.2 million Americans, or 10.5% of the population (1). Atherosclerotic cardiovascular disease (ASCVD) is the leading cause of death in individuals with diabetes. In 2017, the morbidity and mortality from ASCVD resulted in an estimated $37.3 billion in healthcare costs in diabetes associated expenditures (1). More than 50% of patients with type 2 diabetes (T2D) have pre-existing CVD at the time of diagnosis (2). But these early CVD prevalence data are unclear in patients with type 1 diabetes (T1D) (3), where mortality from coronary artery disease is approximately 3- to 10- fold higher than in the general population (2).

**Figure 1:**
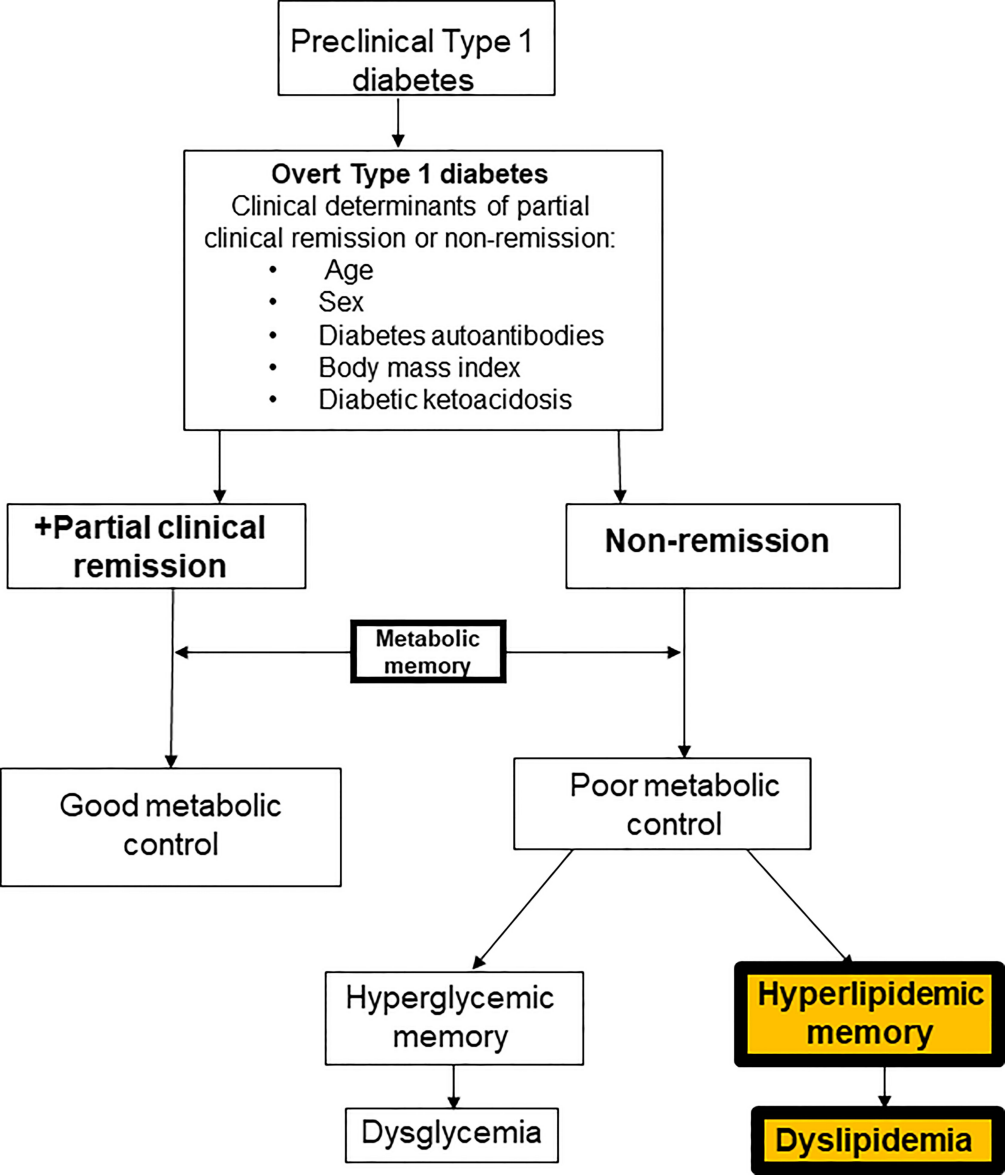
A composite scheme of the dichotomy of subjects with type 1 diabetes based on the history of partial clinical remission into remitters and non-remitters and the role of metabolic memory on long-term metabolic parameters. The theory of *hyperglycemic* memory explains the dysglycemia in non-remitters while the theory of *hyperlipidemic* memory explains the dyslipidemia in non-remitters.

Despite the strong correlation between ASCVD and diabetes mellitus, the underlying mechanisms remain poorly understood (4), especially in T1D where 50% of the subjects undergo partial clinical remission (PR) or honeymoon phase following the diagnosis. However, the impact of PR on the earliest lipid phenotypes in adults with diabetes mellitus is not known (5). Though PR has been reported to modulate the degree of early-phase dyslipidemia (6), mid-term microvascular disease risk (7), and long-term ASCVD risk (8), no prior study in adults (5) has directly compared the earliest phenotype of lipid-based ASCVD risk between subjects with T2D and T1D, after stratifying the T1D subjects into remitters and non-remitters based on their PR history. Such studies are important to establish the prevalence of dyslipidemia in T1D. These studies will also help to characterize some yet unexamined contributors to diabetic dyslipidemia in children and adults with diabetes mellitus, such as the role of hyperlipidemic memory on subsequent lipid phenotypes (**Figure 1**).

### A Literature Review of Current Knowledge in PR

The Diabetes Control and Complications Trial reported a protective role for C-peptide on vasculature in remitters or patients with T1D who had residual β-cell function (8). The Medalist study (9) reported that among adult patients with T1D for >50 years, there is a cohort that still produced insulin, and that this cohort had better glycemic control and lipid profile when compared to their peers. The T1D Exchange study (10) of 919 showed that a great proportion of children and adult patients with T1D were still producing insulin. This study found that residual C-peptide remained 3-5 years after diagnosis in 78% of participants who were diagnosed at >18 years and 46% of those diagnosed at <18 years. Additionally, they found that 6% of subjects with childhood onset-, and 16% of those with adult-onset T1D had residual C-peptide at >40 years after their diagnosis. Despite these landmark findings, there are very sparse data on the characterization of early-phase, post-diagnostic lipid phenotypes in remitters and non-remitters (6) across the lifespan in both children and adults to form a foundational basis for extrapolations to the clinical significance of PR with respect to ASCVD. A review of current literature on dyslipidemia in children and adolescents with T1D shows no consensus on their lipid phenotype, and it is believed that a lack of stratification of subjects by PR history may have confounded these results (11–14). Similarly, a review of the literature in adults subjects with diabetes mellitus showed that while the risk factors for ASCVD are well established in T2D (1), they are less clear in those with T1D (3, 4).

The lack of a detailed analysis of the degree of dichotomy in early lipid phenotypes in T1D subtypes: remitters and non-remitters, and the assumption that subjects with T2D have worse lipid profiles than those with T1D have hindered a thorough assessment of the intrinsic disparities in lipid phenotypes in T1D (1, 3, 4).

### Poor Characterization of ASCVD Risk in T1D

Risk factors for ASCVD are well established in T2D (1), but not in T1D (3, 4). Current knowledge indicates that HbA1c, diabetic nephropathy, hypertension, and dyslipidemia are important risk factors for ASCVD in adults with established T1D (15). However, the phenotype of the earliest ASCVD risk profile at the time of diagnosis of T1D compared to T2D, and the cardinal role of PR on early lipid phenotype in T1D, which presages later ASCVD risk status, are not fully characterized.

Diabetic dyslipidemia, the major link between diabetes mellitus and ASCVD, occurs in the setting of low HDL-cholesterol (HDL-C), high fasting and/or postprandial triglycerides (TG), average to high LDL-C, and predominantly small dense LDL-C particles (16). Elevated non-HDL-C correlates with 99% increased CVD risk for patients with T2D (17) where the classic lipid abnormalities are characterized by elevated TG, small dense LDL-C, and low HDL-C (18). Similarly, CVD risk in T1D is predicted by total cholesterol/HDL cholesterol, and non-HDL cholesterol but not LDL-C (19). The American Diabetes Association (ADA) and the European Association for the Study of Diabetes (EASD) recently updated their position statements on the management of T2D in adults to include additional focus on CVD risk factor management (4), but the recommendations for T1D are vague (4) as the ADA admits that very little clinical trial evidence exists for patients with T1D of any age to issue any meaningful recommendations (1). The current ADA guidelines (1), derived mainly from T2D data, recommend an initial screening for dyslipidemia in adults with diabetes mellitus at the time of diagnosis or at the initial clinic visit, and then to initiate interventions such as intensification of lifestyle modification and optimization of glycemic control for patients with elevated triglyceride levels (≥150 mg/dL) and/or low HDL cholesterol (<40 mg/dL for men, and <50 mg/dL for women). This is based on the concept of the atherogenic index of plasma, denoted by TG/HDL where an atherogenic index of >2 is related to CVD (20, 21).

### C-Peptide Physiology in Relation to Partial Clinical Remission (PR) in Children With Type 1 Diabetes, and the Early Lipid Changes in Type 2 Diabetes Compared to Controls

There is no consensus on the mechanism of dyslipidemia in children with either T1D or T2D. Available reports have differed on the clinical patterns of dyslipidemia in both diseases, as well as the proposed mechanism(s) for the early-phase dyslipidemia in either disease. A key limitation of previous studies was the *lack of consideration* for the role of the honeymoon phase of T1D, also known as partial clinical remission (PR) on the early changes in lipid profile in patients with T1D despite reports that remission status confers special CVD risk protection to youth (6) and adults (8) with T1D. The principal marker for the honeymoon phase or PR is stimulated serum C-peptide concentration (22), and its clinical surrogate marker is the insulin-dose adjusted A1c (22), a functional marker that incorporates both A1c and total daily dose of insulin to determine PR status. C-peptide is a 31 amino acid peptide that is co-secreted with insulin (23). It’s longer half-life of 31 minutes compared to 4 minutes for insulin, makes it a veritable tool for the confirmation of endogenous insulin production and secretion (24, 25). C-peptide has no known receptor, but has been reported to reduce diabetes-related complications such as neuropathy, retinopathy, nephropathy through various hypothesized mechanisms (23, 25–28) which are linked to its regulation of cell proliferation and apoptosis *via* its association with inflammatory mediators such as nuclear factor kappa B, tumor necrosis factor alpha, and protein kinase-C (29). However, the influence of C-peptide on the complications of either T1D or T2D through its impact on early lipid changes in either T1D or T2D has not yet been fully investigated.

### Type 1 Diabetes: The Limitations of the Current Hyperglycemic Theory

PR is a characteristic feature of T1D, which is a disorder of persistent hyperglycemia resulting from autoimmune destruction of the pancreatic β-cells (30, 31). PR often follows the diagnosis of T1D, and this phase is marked by an increased functionality of the surviving β-cells with attendant increased endogenous insulin production (32, 33). Subjects who experienced PR are designated as remitters and those who did not are designated as non-remitters. PR typically lasts for 3-12 months (22), however, recent studies have shown evidence for C-peptide production, and thus residual β-cell function, at more than 5 years following the diagnosis of T1D (34). During PR, C-peptide is co-secreted with insulin from pancreatic β-cells and this residual C-peptide may act as a surrogate marker of residual β-cell function. In physiologic concentrations, C-peptide acts to improve both microvascular blood flow and microvascular endothelial function through the release of endothelial nitric oxide (35). Following the diagnosis of T1D, serum C-peptide concentration undergoes an initial exponential fall followed by a stable phase of decline that may last for several years (34). The presence of residual endogenous insulin secretion in patients with T1D has been linked to reduced risk for severe hypoglycemia (36, 37), development of diabetic retinopathy (38), promotion of statural growth in prepubertal children (39) and a sustained improvement in long-term glycemic control (7, 8). Conversely, the non-remitters experience chronic hyperglycemia from the time of diagnosis (5, 7). This initial phase of chronic hyperglycemia has been associated with long-term complications of diabetes mellitus, regardless of whether glycemic control improved much later in the history of the disease (40). This phenomenology of diabetes complications arising from initial chronic hyperglycemia has been christened the theory of hyperglycemic memory (41). Recent studies show that there are non-glycemic aspects to this phenomenon, and most of these factors are yet to be fully characterized (40). As a result, some investigators now refer to this phenomenon as the glyco-metabolic theory (40). It is generally believed that the mechanisms that lead to the glyco-metabolic memory are *interdependent* and act simultaneously. The four mechanisms currently proposed are oxidative stress, generation of advanced glycation end-products, chronic inflammation, and epigenetic changes (40). ***However, none of the studies in this field has examined the initial post-diagnostic lipid phenotypes in these patients to determine whether a dichotomy exists in the lipid parameters, and whether non-remission is associated with both hyperglycemia and hyperlipidemia.*** Therefore, the theory of *hyperglycemic* memory has limited application as it does not explain the glycemic-independent dichotomy in early lipid phenotypes that presages subsequent differences in ASCVD risks and diabetes-related complications. A theory of *hyperlipidemic* memory, on the other hand, aptly explains this dichotomy and provides the necessary framework to understand the differences in lipid phenotypes between remitters and non-remitters on one hand, and between those with T1D or T2D on the other. This new paradigm is supported by a longitudinal study that reported a significantly reduced risk for chronic microvascular complications at 7-year follow up in young adults who experienced PR (7), as well as another study showing favorable lipid phenotype 5 years after the diagnosis of T1D in children who experienced PR (42).

### Type 2 Diabetes

T2D, on the other hand, is a complex genetic disorder marked by persistent hyperglycemia as a result of a combination of increased β-cell apoptosis and insulin resistance (43, 44). There are significant pathophysiological, prodromal, and post-diagnostic differences between T1D and T2D that play important roles in their early lipid phenotypes. Sagesaka et al. reported that glucose dysregulation precedes the actual diagnosis of T2D by >10 years (45) in adults, while Lebovitz et al. reported that β-cell dysfunction in adults precedes clinical diagnosis of T2D by 12 years (46). In contrast, the diagnosis of T1D is often followed by the honeymoon phase or PR which largely determines the risks for early-phase dyslipidemia (6), mid-term microvascular disease risk (7), and long-term CVD risk (8). We have directly compared lipid-based CVD risk profile between T2D and T1D patients based on the PR history of the T1D cohort (5, 47).

Furthermore, the assumption that early dyslipidemia in children and adolescents with T2D is due to increased insulin resistance (IR) has not been tested by comparing their lipid parameters to those of non-remitting subjects with T1D, who do not have significant IR. Such a comparison will likely determine the role of IR on early lipid changes in children with diabetes mellitus; and may lead to a unified mechanistic model for dyslipidemia in those with diabetes mellitus. We have presented evidence from our studies showing that PR is the primary determinant of early lipid phenotype in pediatric and adult T1D, while other determinant such as BMI, sex, race/ethnicity, and glycemic control play only secondary roles (5, 6, 42, 48) as detailed below.

## Rationale for Investigations on the Theory of Hyperlipidemic Memory in Pediatric Type 1 Diabetes: Lack of Consensus on Early Lipid Phenotypes in Children and Adolescents

### Background

Cardiovascular disease (CVD) is the leading cause of mortality in patients with diabetes mellitus (49). Dyslipidemia and atherosclerosis, which begin in childhood and adolescence^29,30^, are primary contributors to the increased CVD risk in patients with T1D (50, 51). A pediatric study reported that 25% of youth with T1D have progressive and persistent dyslipidemia and increased arterial stiffness (11), while another found a positive association between increased arterial stiffness and total cholesterol (TC), LDL-cholesterol (LDL-C), and HbA1c (12). There is, however, no consensus on either the patterns of early lipid changes, or the mechanism of these changes in children and adolescents with either T1D or T2D.

### Lack of Consensus on the Patterns of Early Dyslipidemia in T1D and T2D

Hanks and co-workers conducted a comparative analysis of primary lipid parameters in overweight/obese youth with either T1D or T2D and found no significant differences in the concentrations of the primary lipid parameters between the groups (52). However, Rodriguez et al (53) reported a higher prevalence of CVD risk factors in youth with T2D compared to T1D, while Kim et al (54), in a 10-year longitudinal study that examined overall CVD risks between T1D and T2D reported increasing prevalence of elevated waist circumference in patients with T2D as its primary finding. Kim et al (54) found no significant longitudinal changes in the prevalence of other risk factors, including lipid concentrations, throughout the period of observation. Thus, there is no consistent pattern for early dyslipidemia in children and adolescents with T1D and T2D that could form the basis for a unified mechanistic theory of dyslipidemia in children with diabetes mellitus.

### Lack of Consensus on the Mechanism of Early Dyslipidemia in T1D and T2D

There is equally no consensus on the mechanisms for early-phase dyslipidemia in youth with either T1D or T2D due to the disparate conclusions from published studies (55–59). Maahs et al. and other investigators had suggested that adiposity and IR (54, 58, 59) played a central role in the pathogenesis of dyslipidemia in children with diabetes mellitus. A cross-sectional study from the SEARCH Group (60) in the US also reported a relationship between increasing A1c and dyslipidemia in subjects with either T1D or T2D, but a UK-based longitudinal study in subjects with T1D found no such association (55). In support of the findings from the UK-based study (55), Katz et al, in a longitudinal retrospective cohort study of subjects with T1D found that changes in HbA1c and BMI z scores had minimal impact on LDL-C and non-HDL cholesterol (13). Though Shah et al (11, 13) and others reported a significant relationship between poor glycemic control and dyslipidemia in T1D (11, 13), others found an inconsistent pattern of correlation of lipid concentrations and HbA1c (61), or no correlation at all (62). Snell-Bergeon and others reported that systemic inflammation (57, 63) and glycemic control (55–57) play only a marginal role on early lipid changes in either T1D or T2D. Therefore, there is no consensus on early lipid phenotypes in children with diabetes mellitus. It is possible that a lack of consideration for the role of residual β-cell function or honeymoon phase in their respective T1D cohorts (52–54, 64) could have led to the disparate conclusions. None of the above studies explored the differences in lipid profiles based on patients’ remission status, except in the case of Redondo et al (65) whose findings were confounded by the underestimation of PR by insulin dose adjusted A1c (IDAA1c) in ethnic minority youth (66).

### Pathway to a Consensus on the Mechanism and Pattern of Early Dyslipidemia in T1D and T2D

The stratification of subjects with new-onset T1D by PR status is critical to ensure meaningful comparisons of lipid parameters for valid results (11–14). For instance, it is unknown whether the study that reported progressive and persistent dyslipidemia (11) contained a higher proportion of non-remitters, while the study that reported only a modest effect of HbA1c and BMI on lipid parameters (13) had a higher proportion of remitters. The fact that non-remitters make up >50% of children and adolescents with new-onset T1D (67, 68) makes it crucial to stratify subjects based on their PR history in all research studies assessing lipid parameters in patients with T1D. This will ensure proper stratification of risk for CVD by PR and may lead to the accumulation of data to designate non-remission as a non-modifiable risk factor for ASCVD in patients with T1D.

## Investigation of the Role of Partial Clinical Remission on Early Lipid Phenotypes in Pediatric Type 1 Diabetes

### Clinical Studies Demonstrating the Role of PR on Early Lipid Phenotypes in T1D

**Figure 2:**
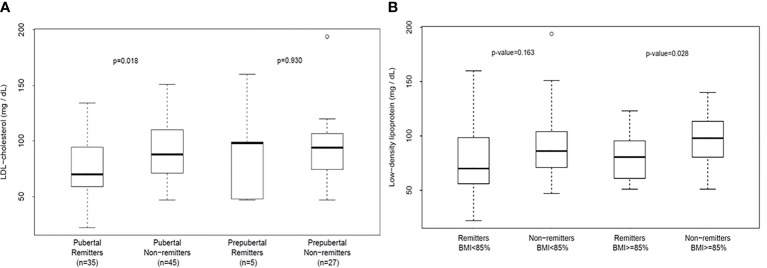
**(A)** (left panel) and **(B)** (right panel): **(A)** shows that analysis of the differences in serum low-density lipoprotein cholesterol (LDL-C) concentration in the first 4-5 years of type 1 diabetes stratified by both pubertal and remission status. LDL-C concentration was similar in prepubertal remitters vs. non-remitters (p=0.930) but was significantly lower in remitters in puberty compared to the non-remitters in puberty (p=0.018) after adjusting for age and duration of diabetes (6). **(B)** depicts the analysis of the changes in serum low-density lipoprotein cholesterol (LDL-C) concentration in the first 4-5 years of type 1 diabetes stratified by both body mass index and remission status. Serum LDL-C was similar between the normal-weight remitters and non-remitters: 79.0 ± 32.8 mg/dL vs. 89.8 ± 27.5 mg/dL, p=0.17. In contrast, LDL-C was significantly lower in remitters among the overweight/obese cohort, 78.5 ± 21.1 mg/dL vs 95.6 ± 24.2 mg/dL, p=0.028 (6).

To explore the role of PR on early lipid changes in children, we conducted a longitudinal retrospective cohort study of 123 children and adolescents with T1D of 5-year duration (6). The subjects’ mean age was 11.9 ± 2.9 years, and the cohort consisted of 55 male subjects and 68 female subjects. There were 44 remitters and 79 non-remitters. A timeline of 4-5 years after diagnosis was chosen in concert with the American Diabetes Association (ADA) recommendation to initiate screening for diabetes complications in children either at the inception of puberty or 4-5 years after diagnosis (69) as it was previously believed that there was minimal risk of dyslipidemia during the prepubertal years (69). This study excluded children with dyslipidemia or a family history of lipid abnormalities. The results showed that children and adolescents who experienced PR had significantly lower mean LDL-C 4-5 years after the diagnosis of T1D compared to their peers who did not experience PR (6), after controlling for age, puberty, glycemic control, and adiposity [**Figure 2** (6)]. The significantly lower LDL-C in remitters was rather striking as a greater proportion of the remitters were in puberty 4-5 years after the diagnosis of T1D compared to the non-remitters. This was the first report to provide critical and objective evidence of an early lipid-based cardiovascular protection by PR in children with T1D.

To validate these results and confirm that these lipid-based findings were not influenced by normal, physiological, puberty-mediated changes in lipid concentrations in youth, we conducted a follow-up study that compared the T1D cohort to age-matched controls.

## Investigation of the Role of Pubertal Maturation on Early Lipid Changes in Remitters, Non-Remitters, and Controls

The primary rationale for the second study (8) was to determine whether pubertal maturation impacts physiological changes in lipids in children and adolescents with T1D by comparing the T1D cohort to controls to investigate whether subjects with T1D showed similar lipid changes as controls during puberty. This is crucial as the origins of the dichotomy in CVD risk in adults with T1D are rooted in childhood (6–8), but the exact mechanism and point of divergence from normal in CVD risk are not known. The secondary rationale was to either support or disprove the unverified hypothesis that youth with T1D did not experience a reduction in TC, LDL-C, and non-HDL during puberty (62), a phenomenon that occurs in healthy children and adolescents without T1D (70, 71).

**Figure 3:**
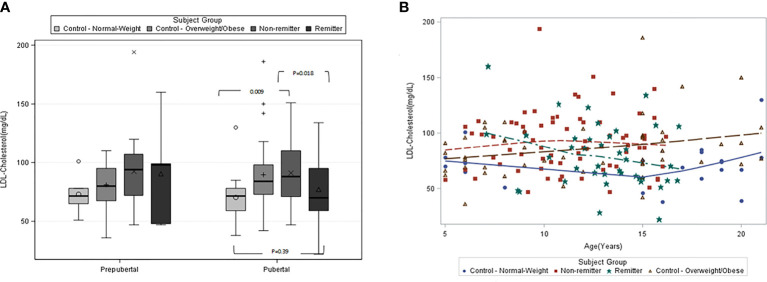
**(A)** (left panel) and **(B)** (right panel): **(A)** is the box plots of low-density lipoprotein cholesterol (LDL-C) concentration stratified by pubertal status in controls and subjects with type 1 diabetes. Among the pubertal cohort, LDL-C was significantly higher in the non-remitters compared to the remitters (p=0.018), significantly higher in the non-remitters compared to the normal-weight controls (p=0.009). LDL-C was significantly higher in the overweight/obese controls compared to the normal-weight control (p=0.033), but similar between the normal-weight controls and remitters (p=0.39) (42). **(B)** is the scatterplot of the comparison of the patterns of low-density lipoprotein cholesterol (LDL-C) in controls and subjects with type 1 diabetes. Both the remitters and the normal-weight controls demonstrated lower LDL-C during puberty, while the overweight/obese controls and the non-remitters did not (42).

This study (42) included 194 subjects consisting of 71 controls of age 12.9 ± 1.3y and 123 subjects with T1D stratified into remitters (n=44, age 13.0 ± 0.8y) and non-remitters (n=79, age 11.2 ± 0.6y). PR was defined as insulin-dose adjusted HbA1c of ≤9 (22). Pubertal status was determined by Tanner staging of breast development in girls, and testicular volume in boys. We found that among the pubertal cohort, LDL-C was significantly higher in the non-remitters compared to the remitters, 91.1 ± 25.6mg/dL vs 77.2 ± 25.8 mg/dL, p=0.018; and the normal-weight controls, 91.1± 25.6 mg/dL vs 70.4 ± 22.9 mg/dL, p=0.009; but was similar between the overweight/obese controls and non-remitters, 89.7 ± 28.9 mg/dL vs 91.1± 25.6 mg/dL, p=0.81, and similarly between the normal-weight controls and remitters, 70.4 ± 22.9 mg/dL vs 77.2 ± 25.8 mg/dL, p=0.39 [**Figure 3A** (42)]. Both non-HDL-C and TC showed similar patterns as the LDL-C.

This was the first study to characterize the natural pattern of lipid profiles in children and adolescents with T1D as they traverse through puberty based on stratification by remission status, while comparing their lipid profiles to healthy peers [**Figure 3B** (42)].

There were 3 novel findings from this study. The first finding was that remission status largely determines the pattern of lipid concentrations in youth with T1D during puberty such that children with T1D who experienced the honeymoon phase or PR showed similar reductions in LDL-C, TC, and non-HDL-C as do normal-weight, healthy children without T1D (71), while non-remitters did not. The study further showed that the *timing* of the onset of the *dichotomy* in lipid profiles, and consequent CVD risk, in youth with T1D occurs between ages *11-12* years for LDL-C, TC, and non-HDL cholesterol. This age definition for lipid phenotype dichotomy is consistent with the timing of the onset of physiologic reduction in LDL-C, TC, and non-HDL during puberty in children without diabetes mellitus (72).

The stratification of the subjects into remitters and non-remitters was central to these findings, and thus showed that the lack of consensus on lipid phenotypes in children and adolescents with T1D from earlier studies could have derived from the non-stratification of subjects by PR history (11–14).

The second novel finding was that remitters have an intrinsic protection against adiposity-driven dyslipidemia, and this protection was absent in non-remitters as demonstrated by the significantly elevated LDL-C in overweight/obese non-remitters compared to overweight/obese remitters during puberty [**Figure 2** (6)]. This is consistent with the earlier report that residual C-peptide has a vascular protective function (8) and could protect remitters from early-phase anatomic changes in vasculature caused by dyslipidemia.

The third novel finding was that overweight/obese children without T1D do not experience the classic physiologic reduction in LDL-C, TC, and non-HDL that was described by Eissa et al (71) in healthy children and adolescents during puberty. This finding is important because Eissa et al (71) did not stratify their subjects by BMI status, and so were unable to detect this secondary effect that is largely driven by adiposity. The detection of a dichotomy in lipid phenotypes in normal children based on their BMI status led us to the hypothesis that the increased levels of LDL-C, non-HDL, and TC in the overweight/obese children and adolescents might be due to the presence of non-functional C-peptide in their circulation, similar to the concept of insulin resistance.

This study highlights the central role of C-peptide physiology on early lipid changes in children and adolescents. It is generally believed that the mechanism for the reduction in LDL-C, TC, and non-HDL-C during puberty is related to the effect of sex hormones on lipoprotein metabolism, specifically changes in alpha and beta lipoproteins (72). We proposed that this puberty-mediated reduction in the concentrations of LDL-C, TC, and non-HDL-C could be attenuated or abolished by increased insulin resistance (73) as reported in our overweight/obese cohort, due to the non-functional C-peptide effect. In contrast, PR appears to facilitate this normal physiologic reduction in LDL-C, TC, and non-HDL-C in youth with T1D. This reduction is however absent in the non-remitters, who lack endogenous insulin or C-peptide activity. This concept of non-functional C-peptide effect was recently confirmed by Mock et al (74) who reported that 55% of youth with new-onset T1D and detectable C-peptide of >300 pmol/L had low insulin sensitivity scores at 14.5 months following the diagnosis of T1D, and thus were not in PR when defined by IDAA1c.

Based on these finding we decided to explore whether a C-peptide mechanistic model or an adiposity model (based on BMI) could explain early changes in lipids in T2D by comparing subjects with T2D, who are classically insulin resistant, to non-remitters who are relatively not insulin resistant, while using the controls and remitters as comparators.

## A Comparative Analysis of the Earliest Post-Diagnostic Lipid Phenotypes in Remitters, Non-Remitters, Type 2 Diabetes, and Controls in Children and Adolescents

The primary rationale for this investigation of the early lipid phenotypes in children and adolescents with either T1D or T2D was to explore the basis for the lack of consensus on the accurate patterns and mechanisms of early ASCVD risk in children and adolescents with either T1D or T2D (52–54, 64). The secondary rationale was to investigate the unproven premise that pediatric patients with T2D have worse lipid profiles than their peers with T1D in the early phases of T1D or T2D. This is important as no prior study had compared early lipid phenotypes in patients with either T1D or T2D after stratifying the T1D cohort into remitters and non-remitters despite reports that remission status confers special CVD risk protection on youth (6) and adults (5) with T1D.

The aim of this investigation was to determine the differences in ASCVD risk, using lipid parameters as surrogates, in children and adolescents with either T1D or T2D at the time of their first lipid assessment, after stratifying the T1D cohort into remitters and non-remitters. The study’s hypothesis was that the remitters and controls would have similar and more favorable lipid phenotype compared to the non-remitters and subjects with T2D.

**Figure 4:**
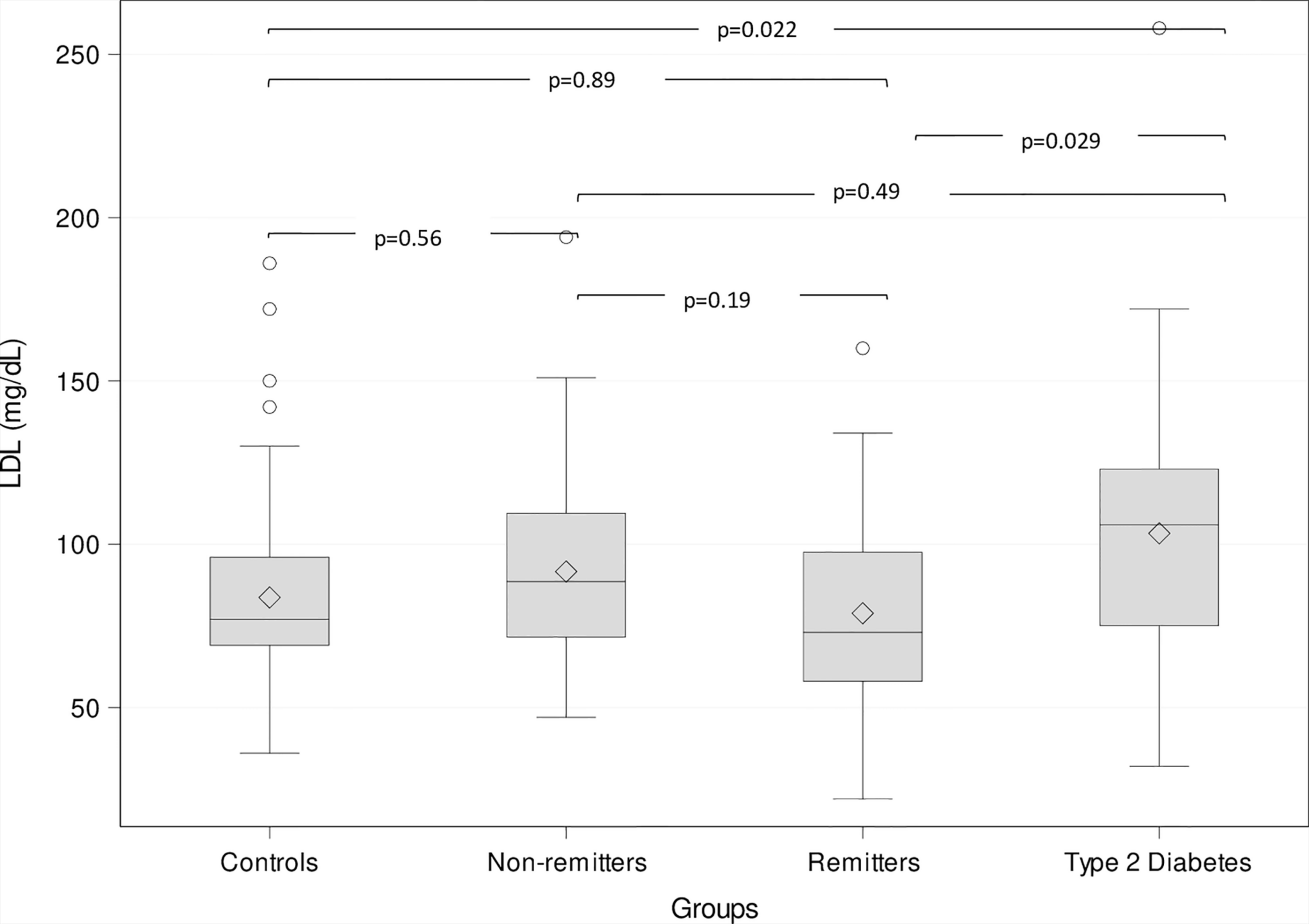
Box plots of the comparison of serum low-density lipoprotein (LDL)-cholesterol (LDL-C) among the groups. The lines through the middle of the boxes represent the median or middle quartile, while the lines at the top and bottom of box represent the upper and lower quartiles, respectively. The upper and lower whiskers represent the scores outside the middle 50%, while the circles represent the outliers. Serum LDL-C was significantly higher in the subjects with type 2 diabetes compared to the controls and the remitters, but similar to the non-remitters (47).

This study (47) included 249 subjects of <21 years consisting of 73 controls, 53 T2D subjects, and 123 T1D subjects stratified into remitters (n=44), and non-remitters (n=79). Partial clinical remission (PR) was defined as insulin-dose adjusted HbA1c of ≤9, and pubertal status was determined by Tanner staging of breasts in girls and testicular volume in boys [**Table 1** **(** 47)]. The results showed that after adjusting for age, sex, BMI, race, and pubertal status, patients with T2D had significantly higher LDL-C compared to the controls (103.1 ± 5.9 mg/dL vs 83.9 ± 3.6 mg/dL, p=0.022), the remitters (103.1 ± 5.9 mg/dL vs 79.1 ± 5.2 mg/dL, p = 0.029), but not the non-remitters (103.1 ± 5.9 mg/dL vs 91.4 ± 4.2 mg/dL, p = 0.49) [**Figure 4** (47)].

**Table 1:** Anthropometric and biochemical characteristics of the subjects (47).

Parameters	Controls n=73	Non-Remitters n=79	Remitters n=44	Type 2 diabetes n=53	*p* value
Age (years)	12.8 ± 5.2	11.3 ± 2.9	13.0 ± 2.5	18 ± 3.1	<0.001
Sex					0.346
• Male (%)	53%	41%	52%	43%	
• Female (%)	47%	59%	48%	57%	
Race					0.001
• White (%)	62%	78%	82%	51%	
• Non-white (%)	38%	22%	18%	49%	
Pubertal Status					<0.001
• Tanner I (%)	37%	38%	14%	0%	
• Tanner II-V (%)	63%	62%	86%	100%	
BMI Status in percentile					<0.001
• Normal-weight (<85^th^) (%)	29%	70%	64%	0%	
• Over-weight/obese (≥85^th^) (%)	71%	30%	36%	100%	
Height z-score	0.2 ± 1.4	-.01 ± 1.2	0.1 ± 0.9	0.9 ± 1.4	<0.001
Weight z-score	1.6 ± 1.3	0.5 ± 1.0	0.7 ± 0.8	2.7 ± .7	<0.001
(BMI) z-score	1.7 ± 1.1	0.63 ± 0.9	0.7 ± 0.8	2.4 ± .4	<0.001
SBP (mm Hg)	111.7 ± 11.8	107.8 ± 11.8	111.3 ± 12.8	122.4 ± 13.1	<0.001
DBP (mm Hg)	69.9 ± 8.9	70.2 ± 7.0	70.6 ± 6.0	76.7 ± 8.1	<0.001
Hemoglobin A1c (%)	N/A	8.8 ± 1.2	8.6 ± 1.5	6.7 ± 1.3	<0.001

BMI, body mass index; SBP, systolic blood pressure; DBP, diastolic blood pressure. N/A, not applicable. Remission status was defined by an insulin-dose adjusted hemoglobin A1c (IDAA1c) of ≤9 (22).

Similarly, T2D patients had significantly higher non-HDL-C compared to the controls (p=0.006), the remitters (p=0.0002), but not to the non-remitters (137.6 ± 7.1 mg/dL vs 111.71 ± 5.0 mg/dL, p=0.053). Total cholesterol was also significantly higher in T2D patients compared to the controls (p=0.0005), the remitters (p=0.006) but not to the non-remitters (183.5 ± 6.6 mg/dL vs 166.2 ± 4.8 mg/dL, p=0.27).

This study showed that after adjusting for confounding variables, the serum concentrations of the primary lipid markers: LDL-C, non-HDL-C, and TC were significantly elevated in children and adolescents with either T2D or the non-remitters, compared to controls and the remitters. This report, which is based on stratification of T1D subject into remitters and non-remitters, *clarifies* the long-standing incongruent results of earlier studies that evaluated lipid phenotypes in children with either T1D or T2D (52–54, 64), and makes the case for the stratification of subjects with T1D into remitters and non-remitters to ensure valid comparisons of early lipid phenotypes in this field.

## A Comparative Analysis of the Earliest Post-Diagnostic Lipid Phenotypes in Remitters, Non-Remitters, Type 2 Diabetes, and Controls in Adults

The rationale for this investigation of the early lipid phenotypes in adults with either T1D or T2D was predicated on the fact that risk factors for ASCVD are well established in T2D (1), but not in T1D (3, 4). This is an important area of study as no prior study in adults has compared early lipid phenotypes in patients with either T1D or T2D after stratifying the T1D cohort into remitters and non-remitters, despite reports that remission status confers special CVD risk protection on patients with T1D (6, 8).

The aim of this study was to investigate the impact of PR on the earliest ASCVD risk phenotype in adult patients with T1D by using factor analysis to quantify and compare the ASCVD risk scores of lipid phenotypes in T1D, T2D and the controls after stratifying the T1D cohort into remitters and non-remitters. We focused this aim primarily on the factor analysis of the American Diabetes Association-recommended initial lipid parameters for the assessment of CVD risk in adults with diabetes namely, TG, HDL-C, and the atherogenic index of plasma, TG/HDL as factor 2; and secondarily on non-HDL-C, LDL-C, TC, TC/HDL-C ratio as factor 1. We hypothesized that the remitters and controls would have similar and more favorable lipid phenotype compared to the non-remitters and subjects with T2D. We further speculated that a proof of this hypothesis could lead to investigations that might generate a generalized theory for the earliest mechanisms of atherogenic lipid profile in patients with either T1D or T2D.

**Figure 5:**
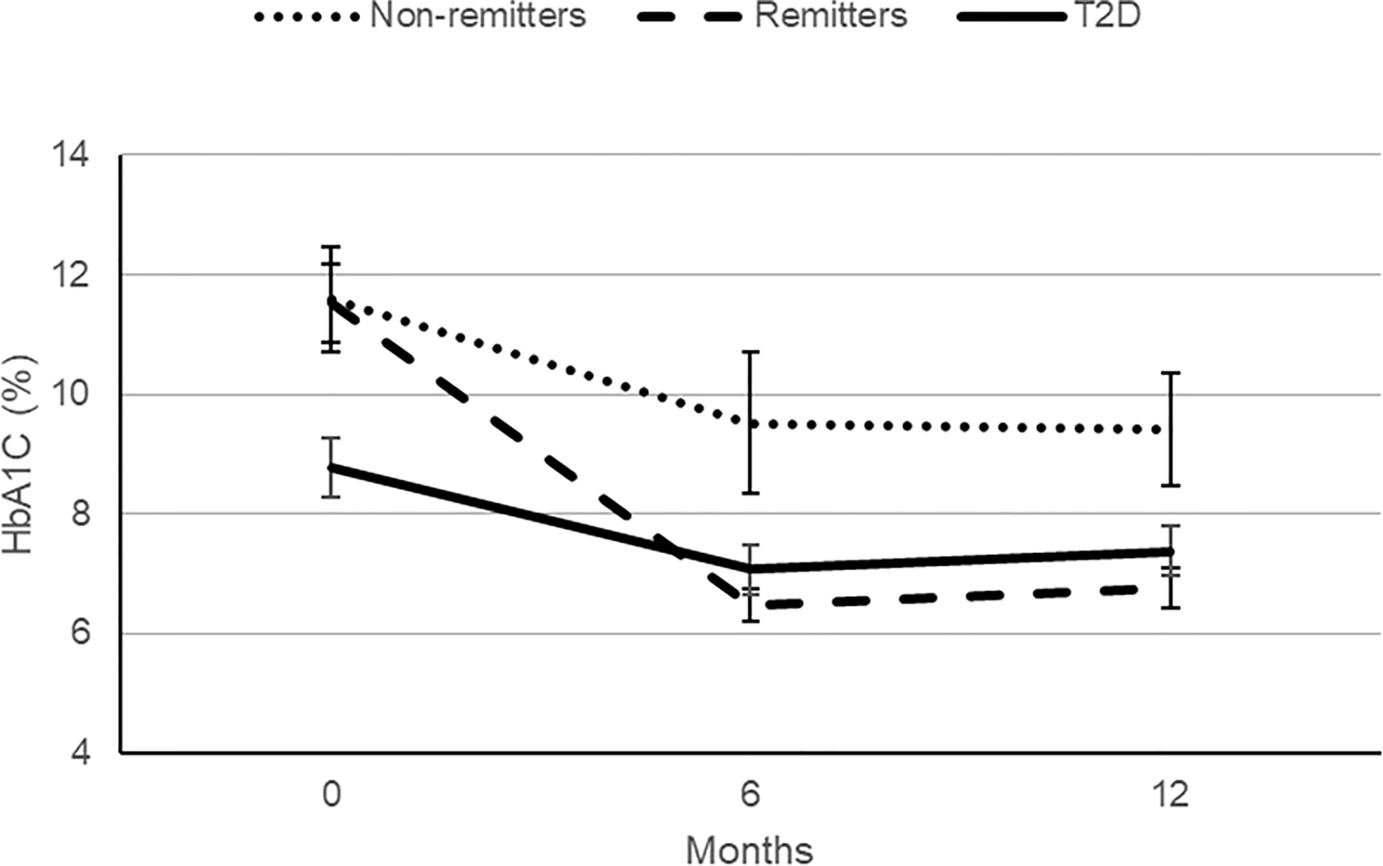
A comparison of the patterns of glycemic control in the remitters, non-remitters, and subjects with type 2 diabetes (T2D) in the first 12 months following the diagnosis of diabetes mellitus [original data from Nwosu et al. (5)].

This was a study of 203 subjects consisting of 40 controls, 77 subjects with T1D, and 86 subjects with T2D. The subjects with T1D were further divided into remitters (n=49) and non-remitters (n=28). The overall mean age was 37.3 ( ± 12.7 SD), with male subjects 51.7% and white subjects 71.3%. Subjects were excluded if they had dyslipidemia, family history of dyslipidemia, or were receiving lipid-lowering medications. **Table 2** **(** 5) shows the baseline anthropometric and biochemical characteristics of the subjects by study group. There were no significant differences in height or gender distribution between the remitters, non-remitters, and subjects with T2D (p=0.44 and 0.91, respectively). Subjects with T2D were older, heavier, and had higher systolic and diastolic blood pressure readings than the subjects with T1D (p<.0001). The non-remitters had significantly higher fasting blood glucose levels (p<.0001). **Figure 5** [original data from Nwosu et al (5)] shows the pattern of glycemic control in the subjects in the first year of the study. The non-remitters had the worst glycemic control in the 12 months of observation.

**Table 2:** Comparison of anthropometric, biochemical, and therapeutic parameters (5) .

Parameters	Controls (n=40)		Remitters (n=49)		Non-remitters (n=28)		Type 2 diabetes (n=86)		ANOVA F-test p value		
	Mean	SD	Mean	SD	Mean	SD	Mean	SD	All 4 groups	3 DM groups	
Age (year)	33.8	11.0	29.7	10.9	31.9	11.0	45.0	10.5	<.0001	<.0001	
Height (cm)	164.9	9.5	170.9	11.8	167.1	8.8	170.4	10.2	0.0450	0.44	
Weight (kg)	74.8	20.2	78.6	17.4	68.6	11.3	104.7	28.7	<.0001	<.0001	
BMI (kg/m^2^)	26.6	6.1	27.0	6.3	25.6	3.2	35.4	9.5	<.0001	<.0001	
SBP (mm Hg)	114.6	17.6	117.5	13.0	113.3	14.0	133.3	17.3	<.0001	<.0001	
DBP (mm Hg)	71.4	13.9	74.5	8.2	70.7	10.7	83.5	10.8	<.0001	<.0001	
FBS (mg/dL)			224	130	425	232	201	116		<.0001	
TC (mg/dL)	155.8	18.6	182.6	72.1	186.2	49.1	192.5	44.9	0.0014	0.62	
LDL-C (mg/dL)	86.1	17.9	100.5	34.8	105.1	32.1	110.9	35.0	0.0011	0.31	
HDL-C (mg/dL)	55.6	12.4	50.5	13.8	46.7	12.8	38.8	9.8	<.0001	<.0001	
TC/HDL	2.9	0.7	4.1	3.9	4.2	1.6	5.2	1.7	<.0001	0.0329	
Non-HDL-C (mg/dL)	100.1	20.3	132.1	74.3	139.5	48.6	153.7	45.6	<.0001	0.11	
TG (mg/dL)	70.4	26.5	120.4	86.0	171.1	168.1	256.6	277.2	<.0001	0.0095	
TG/HDL	1.3	0.6	2.7	2.3	4.2	4.7	7.6	9.5	<.0001	0.0041	
HbA1c at 0 mo (%)			11.6	2.4	11.7	2.5	8.8	2.3		<.0001	
HbA1c at 6 mo			6.5	0.9	9.0	2.1	7.1	1.4		<.0001	
HbA1c at 12 mo			6.8	1.3	9.4	2.4	7.4	1.7		<.0001	
TDD at baseline(units/kg/day)			0.39	0.18	0.51	0.29	0.40	0.18		0.15	
TDD at 6 mo			0.39	0.20	0.70	0.35	0.27	0.19		<.0001	
TDD at 12 mo			0.42	0.21	0.81	0.31	0.33	0.29		<.0001	
Metformin (mg) baseline							1069	501			
Metformin (mg) final							1492	548			
	n	%	n	%	n	%	n	%			
Sex											
Male	12	30.0	28	57.1	15	53.6	50	58.1	0.0224	0.91	
Female	28	70.0	21	42.9	13	46.4	36	41.9			
Race/Ethnicity											
White	26	65.0	44	91.7	18	64.3	56	65.1	0.0051*	0.0022*	
Black	6	15.0	1	2.1	1	3.6	10	11.6			
Asian	3	7.5	0	0.0	1	3.6	4	4.7			
Hispanic	5	12.5	2	4.2	8	28.6	14	16.3			
Other	0	0.0	1	2.1	0	0.0	2	2.3			

BMI, body mass index; SBP, systolic blood pressure; DBP, diastolic blood pressure; TC, total cholesterol; TG, triglycerides; HDL, high density lipoprotein cholesterol; LDL-C, low density lipoprotein cholesterol; HbA1c, hemoglobin A1c; mo, month; TDD, total daily dose of insulin. *p value for white versus others

### Lipid Analysis

#### Individual Lipid Parameters and Ratios

The initial analysis examined the differences in the individual lipid parameters and ratios among the controls, remitters, non-remitters, and T2D subjects. For the individual lipid parameters, the median and the first and the third quartiles were reported to address the skewed distribution of these parameters.

#### Non-HDL-C

**Figure 6:**
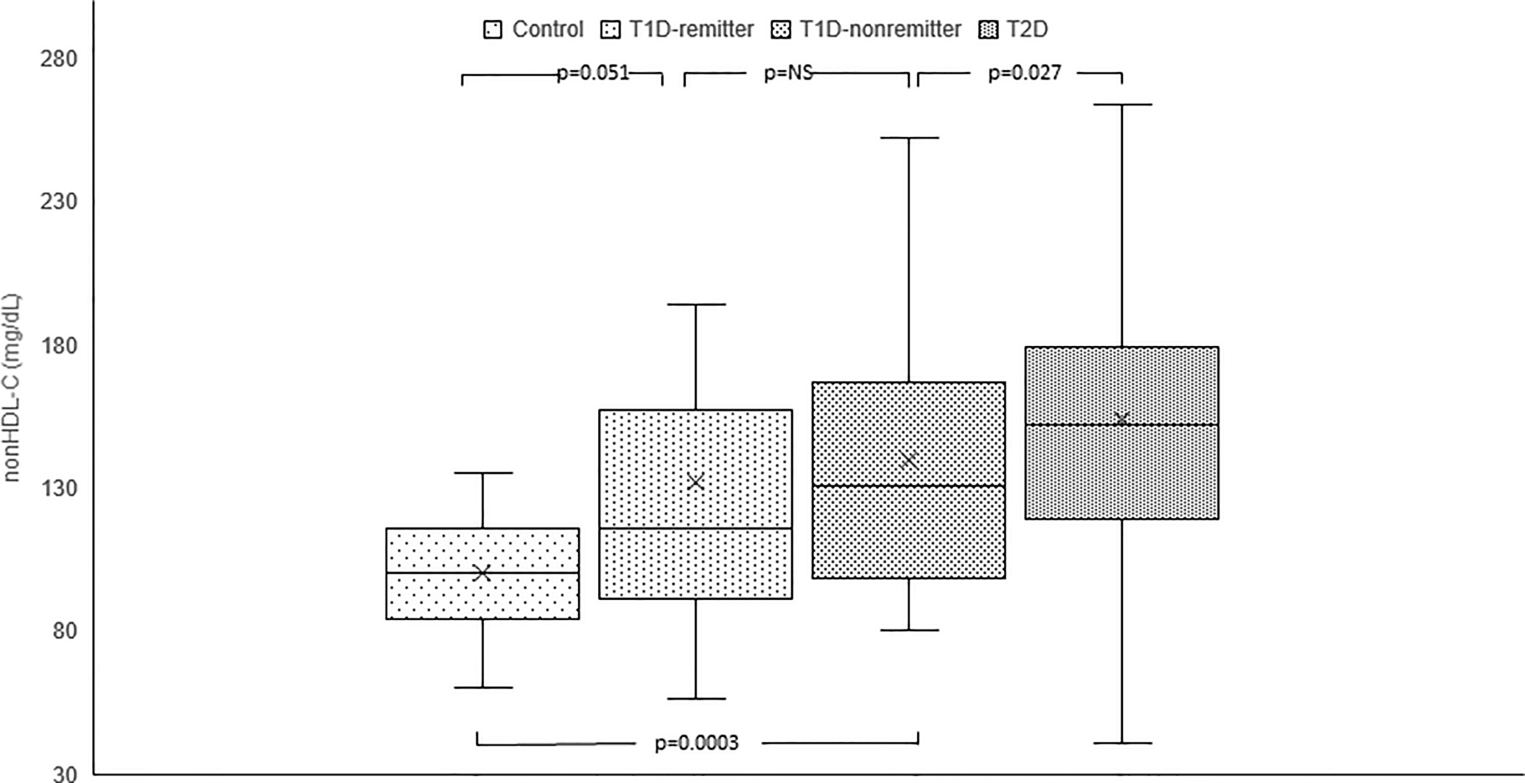
Box plots of early post-diagnostic patterns of non-high density lipoprotein cholesterol (non-HDL-C) in the remitters, non-remitters, and subjects with type 2 diabetes (T2D) compared to controls. The box represents the 50th percent interquartile range, while the ‘x’ represents the mean and the horizontal line within the box represents the median, and the upper and lower whiskers represent 25^th^ percentile above and below the mean, respectively (5).

Serum non-HDL-C was significantly lower in the controls [median=100 mg/dL, Q1-Q3= (84-116)] compared to the subjects with T2D (152 mg/dL, 119-179, p<0.0001), and the non-remitters (131 mg/dL, 100-167, p<0.0001), but was similar to the remitters (116 mg/dL, 92-155, p=0.051). Additionally, non-HDL-C was significantly lower in the non-remitters compared to the subjects with T2D (p=0.027) but was similar between the remitters and non-remitters (p=0.39) [**Figure 6** (5)].

#### TG

Serum TG concentration was significantly lower in the controls (69 mg/dL, 50-88) compared to the subjects with T2D (194 mg/dL, 134-276, p<0.0001) but was similar between the remitters and non-remitters (94 mg/dL, 66-157 vs 107 mg/dL, 82.5-184, p=NS). Though TG was similar between the non-remitters and subjects with T2D (p=NS), it was significantly lower in the remitters compared to the subjects with T2D (p<0.0001).

#### TG/HDL-C

**Figure 7:**
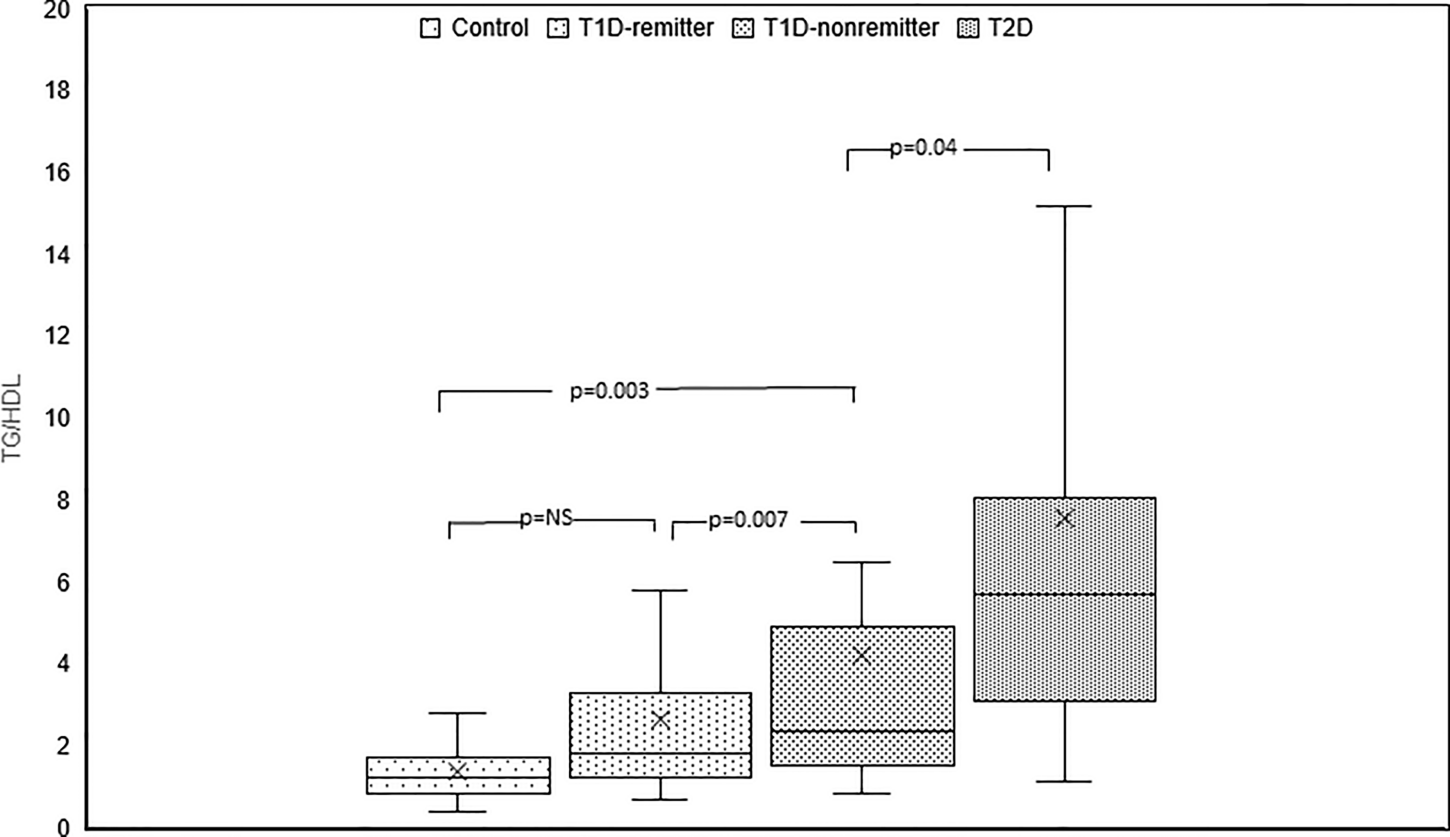
Box plots of early post-diagnostic patterns of triglycerides/high-density lipoprotein cholesterol ratio (TG/HDL) in the remitters, non-remitters, and subjects with type 2 diabetes (T2D) compared to controls (5).

TG/HDL-C ratio was significantly lower in the controls compared to the subjects with T2D (1.2, 0.9-1.7 vs 5.7, 3.1-8, p<0.0001), the non-remitters (1.2, 0.9- 1.7 vs 2.4, 1.5-4.9, p=0.003), but similar to the remitters (1.2, 0.9-1.7 vs 1.8, 1.2-3.3, p=NS). Furthermore, TG/HDL was significantly lower in the remitters compared to the non-remitters (p=0.007) [**Figure 7** (5)].

#### LDL-C

There was a significant difference in serum LDL-C among the 4 groups (p<0.0005). *Post hoc* analysis showed no differences in LDL-C levels among the remitters, non-remitters, and subjects with T2D. However, when compared to the controls, LDL-C was significant higher in the subjects with T2D (p<0.0004), non-remitters (p=0.009), but similar to the remitters (p=0.052).

#### HDL-C

Serum HDL-C was significantly lower in the subjects with T2D compared to the controls (52.5 mg/dL, 45.5-67.0 vs 36 mg/dL, 31.0-45.0, p<0.0001), non-remitters (52.5 mg/dL, 45.5-67.0 vs 49.5 mg/dL, 34.5-56.0, p=0.0217), and remitters (52.5 mg/dL, 45.5-67.0 vs 47.5, 42.0-62.0, p<0.0001). HDL-C was similar between the non-remitters and remitters (49.5 mg/dL, 34.5-56.0 vs 47.5 mg/dL, 42.0-62.0, p=NS)

#### TC/HDL-C

**Figure 8:**
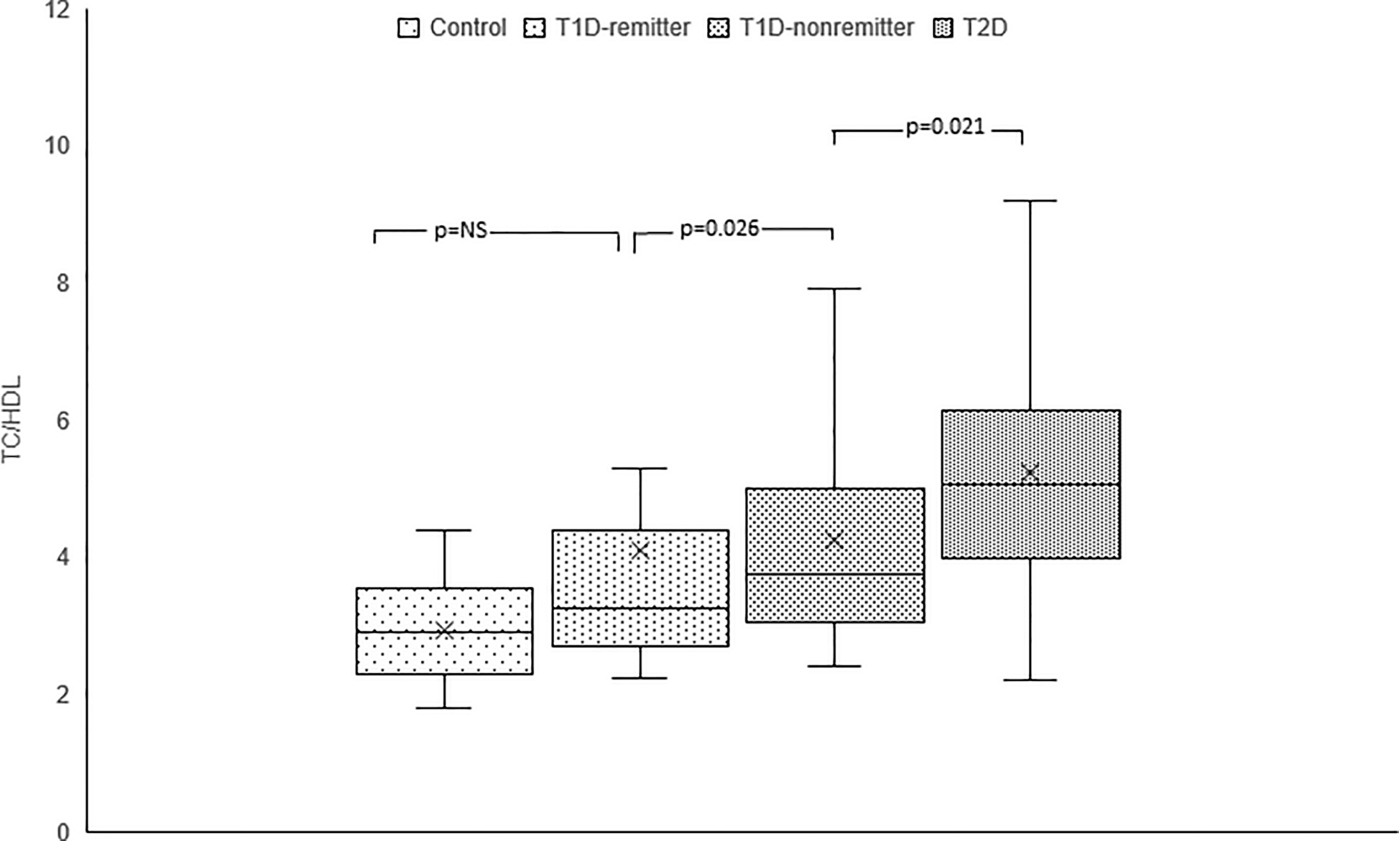
Box plots of early post-diagnostic patterns of total cholesterol/high-density lipoprotein cholesterol ratio (TC/HDL) in the remitters, non-remitters, and subjects with type 2 diabetes (T2D) compared to controls (5).

TC/HDL-C ratio was significantly lower in the controls compared to the subjects with T2D (2.9, 2.3-3.5 vs 5.1, 4.0-6.1, p<0.0001), and the non-remitters (2.9, 2.3-3.5 vs 3.8, 3.1-4.9, p=0.003), but was similar to the remitters (2.9, 2.3-3.5 vs 3.3, 2.7-4.3, p=NS). Additionally, TC/HDL-C was significantly lower in the remitters compared to the non-remitters 3.3, 2.7-4.3 vs 3.8, 3.1-4.9, p=0.026 [**Figure 8** (5)].

#### Factor Analysis of Lipid Parameters

Next, we employed factor analysis to confirm our findings in the individual lipid parameters and to stratify the groups based on their ASCVD risk potential by assigning composite risk scores to the factorized lipid parameters. Factorization of the 7 lipid parameters [**Table 3** (5)] yielded 2 orthogonal factors that jointly explained 89.5% of the total variance in the original 7 lipid parameters with their communalities ranging from 0.74 to 0.99. Based on the structure of the first factor, a composite score was calculated for each subject as a weighted sum of standardized values of the original 7 lipid parameters, with much heavier weights on TC and LDL-C. This composite score was named as TC*LDL.

**Table 3:** Factor analysis of individual lipid parameters and ratios (5).

Factor name	Factor 1	Factor 2	Communality
	TC*LDL	HDL*TG	
TC	0.98	0.12	0.98
LDL	0.97	0.06	0.94
Non-HDL	0.92	0.39	0.99
TC/HDL	0.55	0.78	0.91
HDL	0.08	-0.86	0.74
TG	0.31	0.84	0.80
TG/HDL	0.19	0.94	0.91
%Variance explained	45.3%	44.1%	89.5%

Factors 1 and 2 were derived from factor loading with varimax rotation after adjusting for age, sex, body mass index and ethnicity. TC, total cholesterol; TG, triglycerides; HDL, high-density lipoprotein cholesterol; LDL-C, low-density lipoprotein cholesterol.

**Figure 9:**
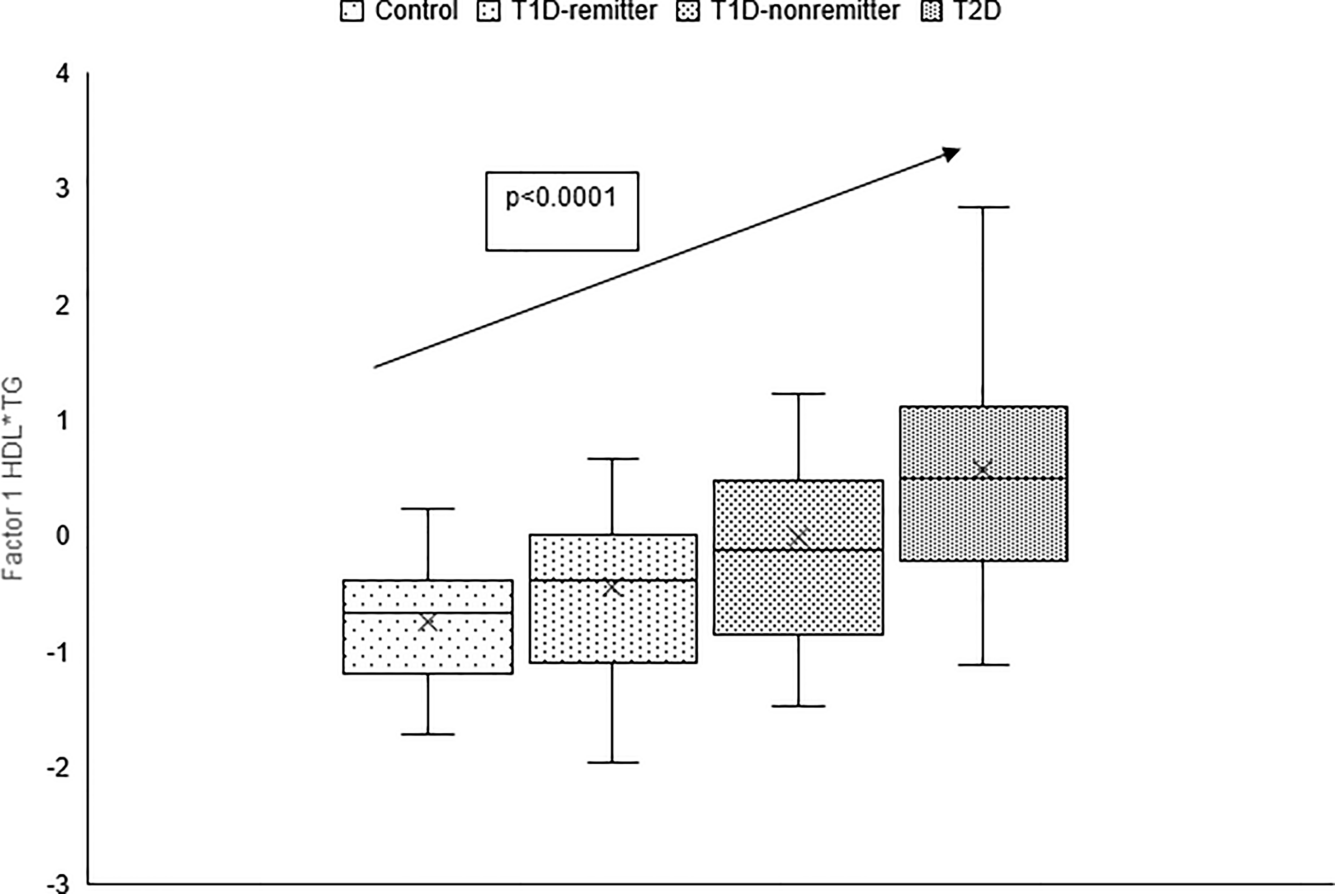
Box plots of the factorial analysis of non-HDL-C, LDL-C, TC, TC/HDL ratio designated as summary factor 1 (TC*LDL) obtained with the factor loading threshold of ≥0.45 in 203 adults. Factor 1 explained 90% of the variance in the original lipid parameters with a linear increase in mean composite scores from controls, remitters, non-remitters, and subjects with type 2 diabetes (p=0.0042) (5).

**Figure 10:**
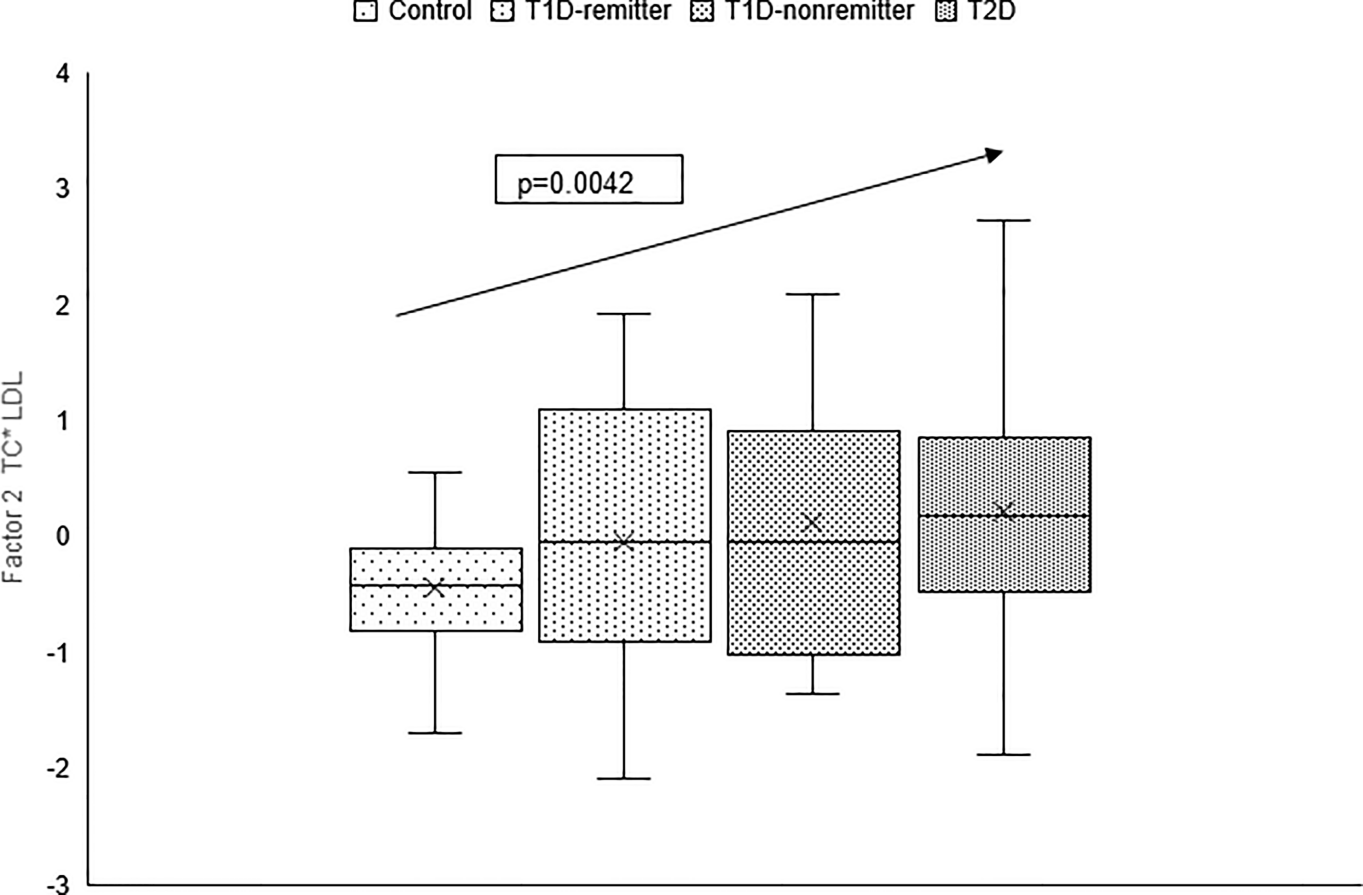
Box plots of the factor analysis of the American Diabetes Association-recommended initial lipid parameters for the assessment of CVD risk in adults with diabetes namely, TG and HDL, and the atherogenic index of plasma, TG/HDL as designated as summary factor 2 (HDL*TG) obtained with the factor loading threshold of ≥0.45 in 203 adults. Factor 2 explained 90% of the variance in the original lipid parameters with a linear increase in mean composite scores from controls, remitters, non-remitters, and subjects with type 2 diabetes (p=0.0001) (5).

**Figure 11:**
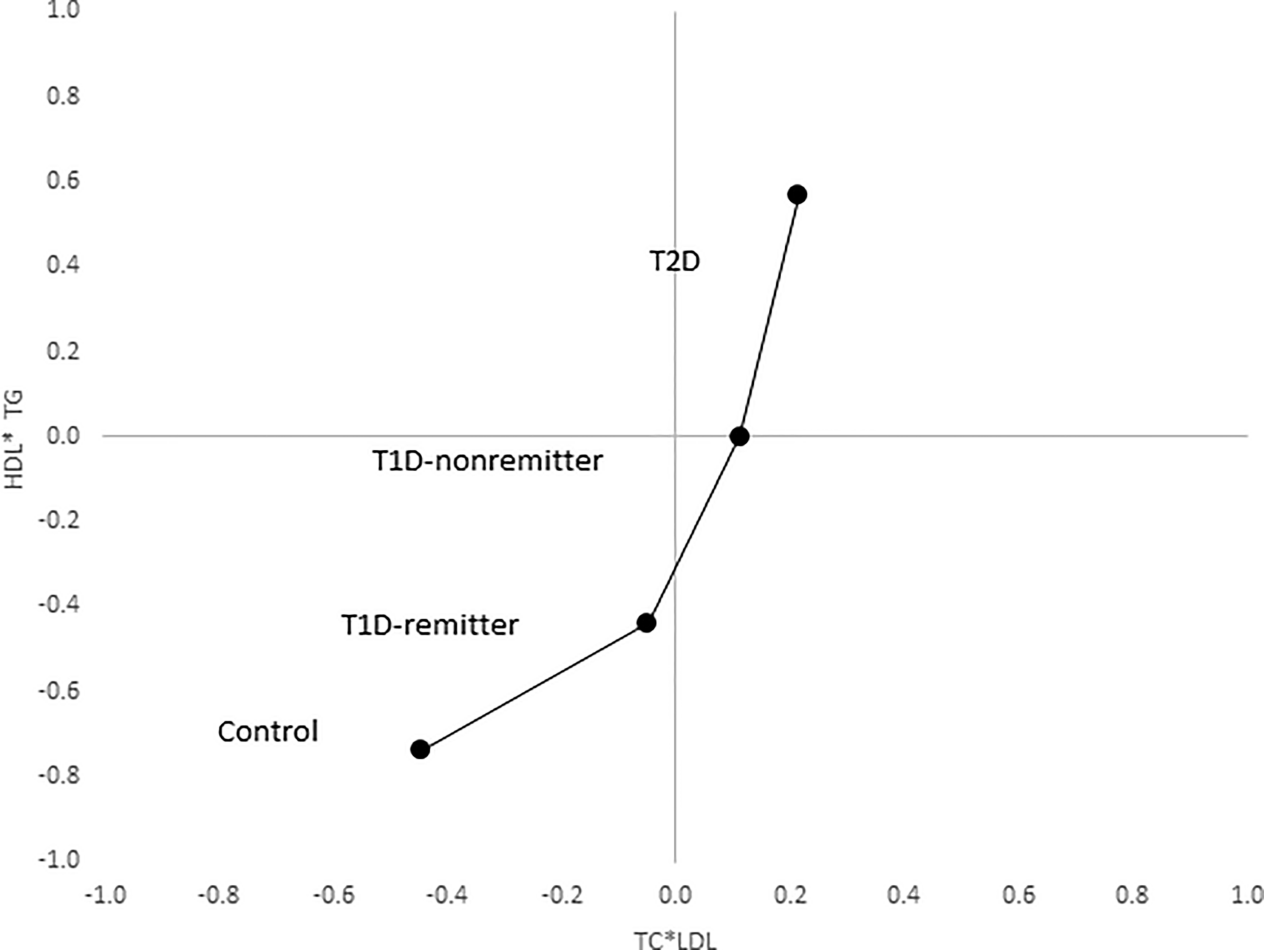
Mean composite score for lipid parameters by factor analysis. Two-dimensional depiction of the mean composite scores of factor 1 (TC*LDL) and factor 2 (HDL*TG) from the factorial analysis of the 7 lipid parameters. Both the controls and remitters are in the low composite risk quadrant, whereas non-remitters and remitters are in the higher risk quadrants. Both factor 1 and factor 2 explained 90% of the variance in the original lipid parameters, p values 0.0042 and <0.0001 respectively. The p-values for linear trends were obtained from linear regression models on composite scores, adjusted for age, sex, ethnicity and BMI (5).

The factor analysis demonstrated a linear increase in the means of both factor 1 (TC*LDL) and factor 2 (HDL*TG) composite scores from the control group to the remitters, non-remitters, and subjects with T2D, p value 0.0042, and <0.0001 respectively as shown in **Figures 9** and **10** (5). This is further illustrated in **Figure 11** (5), a composite two-dimensional plot of factor 1 and factor 2 showing that the controls and remitters occupy the low-risk quadrant, while the non-remitters and subjects with T2D occupy the higher-risk quadrants. These findings in adults confirmed our earlier results in children and adolescents and establish the phenomenon of early dichotomy in lipid parameters in patients with T1D, which we believe, presage the eventual dichotomy in ASCVD risk and prevalence in patients with T1D.

## Discussion

### The Case for PR-Mediated Hyperlipidemic Memory as the Primary Determinant of Early Phenotypes in Both Pediatric and Adult T1D

A comprehensive analysis of the risk factors for dyslipidemia is crucial to the understanding of the central role of hyperlipidemic memory on early lipid phenotypes of both T1D and T2D in relation to other factors associated with dyslipidemia such as glycemic control, BMI, and insulin resistance. The role of glycemic control was examined by Nwosu et al (6) who showed that both remitters and non-remitters have poor glycemic control at the time of diagnosis of T1D, but that glycemic control improves markedly in the remitters and less so in the non-remitters, suggesting that poor glycemic control could lead to dyslipidemia in these patients. However, the fact that the T2D subjects in the follow-up study (47), with less favorable lipid parameters at the time of the diagnosis, had significantly lower mean A1c level of 6.7% compared to the T1D cohort (8.8% for the non-remitters, and 8.6% for the remitters) argues against glycemic control as the principal determinant of early-phase dyslipidemia in children with either T1D or T2D. This finding and previous reports (40) reflect a fundamental limitation of the theory of *hyperglycemic* memory to explain the dichotomy in lipid phenotypes in T1D. Additionally, though BMI is a predictor of dyslipidemia, the presence of normal BMI z-scores in the non-remitters (BMI z-score of 0.63 ± 0.9), despite having a similar lipid profile as the obese T2D patients with a BMI z-score of 2.4 ± 0.4, suggests that increased BMI alone is not the primary etiological factor for increased dyslipidemia in the early phase of T1D or T2D in children. This is further supported by an analysis of the proportion of subjects with dyslipidemia (75) in that study that showed that LDL-C of >130 mg/dL occurred in 7 (13.2%) of the T2D subjects; 6 (7.6%) of the non-remitters; 2 (4.6%) of the remitters; and 4 (5.5%) of the controls. Similarly, TC of >200 mg/dL occurred in 15 (28.3%) T2D subjects; 9 (11.4%) non-remitters; 3 (6.8%) remitters; and 4 (5.5%) controls. This analysis suggests that the non-remitters and the subjects with T2D had a *higher frequency of dyslipidemia* compared to the remitters and controls. Finally, the similarity of early lipid profiles in patients with T2D and the non-remitters, despite their significant differences in BMI z scores, also argues against IR as the sole driving force for dyslipidemia in non-remitters compared to the subjects with T2D. These findings which were confirmed in our adult study (5) establish PR as the primary determinant of early lipid phenotypes in both pediatric and adult T1D.

An alternative theory however could also be entertained. Though the above conclusions are supported by the study data in children and adults, it is possible to advance an alternative conclusion which proposes that the observed differences in the lipid profiles arose from differences in the degree of insulin resistance (IR) in each group such that partial clinical remission served only as a surrogate marker of IR. This conclusion is pertinent as IR occurs in both T1D (74, 76) and T2D; and a recent study by Mock et al. reported that 55% of subjects with new-onset T1D and detectable stimulated C-peptide level of >300 pmol/L had low insulin sensitivity (i.e., high IR) and thus were not in remission when assessed by insulin-dose adjusted A1c (74). Therefore, partial clinical remission, in this alternative theory, may be a marker of IR, with remitters having low IR, and non-remitters having high IR similar to the high IR state in T2D.

### Hyperlipidemic Memory and C-Peptide Physiology: The Synergistic Role of Insulin Sensitivity and C-Peptide Physiology on Early Lipid Phenotypes in Both T1D and T2D

Though C-peptide is considered as an indicator of preserved residual β-cell function, it is a metabolically active molecule (77). Our data suggest that the primary factor leading to the elevations in LDL-C, TC, and non-HDL-C in children and adults with either T1D or T2D is the absolute lack, or the functional absence of the protective role of C-peptide on early lipid changes in diabetes mellitus, such that in the early phases of T1D or T2D in children, there is a functional absence of endogenous C-peptide action in T2D, undetectable C-peptide action in non-remitters, but an active C-peptide effect in the remitters who still produce biologically-active, endogenous C-peptide. Though BMI contributes to dyslipidemia, it does not explain the similarity in lipid phenotypes between the non-remitters who are not obese, and the subjects with T2D, who are obese. However, given the recent study by Mock et al (74) reporting variable levels of insulin sensitivity in youth with similar C-peptide concentrations, it appears that the differences in lipid phenotypes in the various groups could result from a combination of functional C-peptide physiology and differences in insulin sensitivity. Data from our 4 studies suggest that insulin sensitivity and C-peptide physiology articulate a unified mechanistic model for early dyslipidemia across the lifespan in both children and adults with diabetes mellitus.

### Strengths and Limitations of the Studies

The limitations of our studies are related to their retrospective design from which one cannot infer causality, as well as the relatively small sample size for the individual subgroups. The strengths of the study include the careful stepwise progression of the four investigations, the inclusion of pediatric and adult controls and subjects with T2D to clarify the dichotomy in lipid phenotypes in subjects with T1D, the inclusion of longitudinal data, the confirmation of the studies in children with a robust study in adults, and the establishment of composite risk scores for ASCVD by factor analysis.

### Conclusions

We have presented evidence in support of the Theory of Hyperlipidemic Memory based on a series of 4 research studies. These studies which stratified subjects with T1D into remitters and non-remitters and compared their early lipid phenotypes to their peers with T2D as well as controls, demonstrated that across the lifespan: children, adolescents, and adults with T2D and their peers with T1D with no history of PR (i.e., non-remitters) have less favorable lipid phenotype compared to the remitters and controls. These findings strongly suggest the presence of a dichotomy in ASCVD *risk* in subjects with T1D, such that non-remitters have a higher composite risk score for lifelong ASCVD compared to the remitters. The confirmation of this dichotomy in lipid phenotypes between the remitters and non-remitters across the lifespan supports the theory of hyperlipidemic memory whereby the initial hyperlipidemia in non-remitters persists across the lifespan leading to increased risk for ASCVD in this sub-population of subjects with T1D; whereas the imprimatur of PR in the remitters presages a lifetime of favorable lipid profile which has been confirmed in large studies (8). The concept of imprimatur of PR is apt as the metabolic advantages of PR continue long after the end of remission (7). This theory of hyperlipidemic memory explains the principal role of PR history on the early dichotomy in lipid phenotypes in T1D, the subsequent dichotomy in lipid-based ASCVD risks and may provide a new foundation for an early and accurate quantification of ASCVD risk in subjects with T1D across the lifespan.

## Data Availability Statement

The original contributions presented in the study are included in the article/supplementary material. Further inquiries can be directed to the corresponding author.

## Ethics Statement

The studies involving human participants were reviewed and approved by Institutional Review Board of the University of Massachusetts Medical School. Written informed consent from the participants’ legal guardian/next of kin was not required to participate in this study in accordance with the national legislation and the institutional requirements.

## Author Contributions

BN conceived the idea and wrote the manuscript. He is guarantor of the manuscript.

## Funding

This study was funded in part by an investigator-initiated research grant, Grant ID: 1 R21 DK113353-03, to BN from NIDDK, NIH.

## Conflict of Interest

The author declares that the research was conducted in the absence of any commercial or financial relationships that could be construed as a potential conflict of interest.

## Publisher’s Note

All claims expressed in this article are solely those of the authors and do not necessarily represent those of their affiliated organizations, or those of the publisher, the editors and the reviewers. Any product that may be evaluated in this article, or claim that may be made by its manufacturer, is not guaranteed or endorsed by the publisher.
